# Impact of Kangaroo mother care on autonomic cardiovascular control in foetal-growth-restricted preterm infants

**DOI:** 10.1038/s41390-024-03555-z

**Published:** 2024-09-06

**Authors:** Yueyang Tian, Ishmael M. Inocencio, Arvind Sehgal, Flora Y. Wong

**Affiliations:** 1 The Ritchie Centre, Hudson Institute of Medical Research, Melbourne, VIC Australia; 2 Department of Paediatrics, Monash University, Melbourne, VIC Australia; 3 Monash Newborn, Monash Children’s Hospital, Melbourne, VIC Australia

## Abstract

**Background:**

Kangaroo mother care (KMC) is WHO-recommended for low-birth-weight infants, yet its impact on autonomic cardiovascular control in preterm foetal growth-restricted (FGR) infants remains unclear. We hypothesised that KMC would promote autonomic cardiovascular control, benefiting preterm FGR infants with reduced baseline autonomic function compared to appropriate for gestational age (AGA) infants.

**Methods:**

Autonomic control was assessed via heart rate variability (HRV) in low frequency (LF) and high frequency (HF) bands using spectral analysis. Preterm FGR (*n* = 22) and AGA (*n* = 20) infants were assessed for 30-min before and 60-min during KMC. Comparisons were made between FGR and AGA infants; and between infants with baseline HRV below and above median.

**Results:**

Overall, no significant HRV changes were observed during KMC for FGR or AGA infants compared to baselines. Infants with low baseline HRV LF showed increased HRV during KMC (*p* = 0.02 and 0.05 for the entire group and FGR group, respectively). This effect was absent in the AGA group regardless of baseline HRV. Infants with high baseline HRV had decreased HRV during KMC.

**Conclusions:**

Infants with low baseline HRV, suggesting reduced autonomic control, are more likely to benefit from KMC with increased HRV. Further, this effect is stronger in FGR than AGA infants.

**Impact:**

- Kangaroo mother care (KMC) is WHO-recommended for low-birth-weight infants, yet its impact on autonomic cardiovascular control in preterm foetal growth-restricted (FGR) infants is unclear.
- Preterm infants with low baseline heart rate variability (HRV) are more likely to benefit from KMC and increase their HRV suggesting improved autonomic control.
- This effect is stronger in preterm FGR infants than those with appropriate growth for age.

## Introduction

Foetal growth restriction (FGR) refers to the condition where a foetus does not achieve its genetic growth potential due to various maternal, placental, and foetal pathologies.^1^ The principal cause of FGR is uteroplacental vascular insufficiency which leads to chronic foetal hypoxaemia and nutrient deprivation.^2,3^ FGR has been associated with long-term neurodevelopmental impairment and cardiovascular dysfunction.^4,5^ The cardiovascular remodelling following FGR leads to structural changes in vascular wall composition and cardiomyocytes, which predispose to the cardiovascular dysfunction.^5–10^ Furthermore, FGR-related cardiovascular dysfunction may also be due to abnormal development of the autonomic nervous system following the in-utero chronic hypoxia.^11^ FGR infants have demonstrated sympathovagal imbalance with a predominance in sympathetic activity which is strongly linked to hypertension and other cardiovascular risk factors.^12–14^

Both sympathetic and parasympathetic nervous systems are involved in heart rate modulation. Accordingly, autonomic cardiovascular control can be assessed by means of heart rate variability (HRV).^15^ FGR fetuses have been reported to exhibit compromised autonomic cardiovascular control, manifested as altered HRV.^16–18^ In addition, FGR is a major cause of preterm delivery, and prematurity itself has been shown to impair autonomic function.^19,20^ Preterm FGR infants display reduced HRV on the first post-natal day, which could indicate vulnerability to circulatory instability.^21^

Preterm FGR infants often endure prolonged stay in neonatal intensive care units (NICU), facing increased morbidity and mortality compared to preterm infants with weight appropriate for gestational age (AGA).^22,23^ Kangaroo mother parent-infant care (KMC) is recommended by the World Health Organization for the routine care of infants with low birthweight ≤2 kg. KMC promotes parent-infant bonding in the NICU and provides multiple physiological benefits,^24^ including cardio-respiratory stability, improved cardiac output and systemic blood flow, and temperature and stress regulation.^25–29^ Previous studies in preterm infants have reported lesser heart rate decelerations during KMC^24^ and favourable impact on parasympathetic activities.^30,31^

Given the routine use of KMC in the NICU, and its potential positive effects on cardiac function and autonomic regulation in preterm infants, the effect of KMC on autonomic cardiovascular control in preterm FGR infants has yet to be established. Our study aimed to investigate the impact of KMC on autonomic control in preterm FGR infants using HRV analysis, in comparison to preterm AGA infants. We hypothesised that KMC would promote autonomic cardiovascular control, particularly the parasympathetic activities; and this benefit would be more pronounced in preterm FGR infants with reduced baseline autonomic function compared to preterm AGA infants.

## Method

### Subject

Preterm FGR and AGA infants born at ≤32 weeks of gestation age (GA) at Monash Children’s Hospital were studied on or after postnatal day 8 (to allow for postnatal physiology transition). This study was approved by Monash Health Human Research Ethics Committees (HREC 22-0000-571-65599) and parental written informed consent was obtained for all participants.

FGR was identified based on birth weight under the 10th percentile (Growth chart, Pfizer Australia Pty Lt), plus compromised foetal growth and compromised Doppler recordings in foetal systemic arteries. Exclusion criteria included evidence of intrauterine infections, chromosomal abnormalities, major congenital abnormalities (including cardiac diseases) or major brain pathologies such as Grade 3–4 intraventricular haemorrhage and periventricular leukomalacia.

All infants were clinically stable at the time of study, none were being treated with inotropic medications, suffered from meningitis or overwhelming sepsis. All recruited infants received breast milk. The parents had been performing KMC for >3 days at recruitment. Mother and infant were seated in a recliner next to the infant’s incubator. The infant was positioned prone or semi-prone at a 30°–40° angle on the mother’s chest during KMC. Perinatal demographics were retrieved from hospital records.

### Data analyses

Continuous electrocardiograms (ECG), peripheral oxygen saturation (SpO_2_), and respiratory rate as part of routine clinical care were recorded continuously from the neonatal intensive care monitors (Draeger infinity M540 & C500, Drägerwerk AG & Co., Lübeck, Germany). All signals were collected and stored using ICM+ software at 500 Hz (Cambridge Enterprise Ltd, Cambridge, United Kingdom) for 30 min before and 60 min from the start of KMC.

ECG data interrupted by movements and handling were excluded from analysis. Offline analyses were performed (ICM + , Cambridge Enterprise Ltd, UK) to firstly calculate intervals between R waves in the ECG using peak detection algorithm based on the Pan-Tomkins method.^32^ Artifacts like device calibration as well as individual ectopics were automatically deleted using a lower R–R interval threshold of 175 ms to remove spurious beats. Power spectral analyses for HRV were performed on R–R interval series in 120-s time windows that were updated every 10 s.^33^ Fourier transform with set frequency bands was used to compute the spectral power for the R–R series, thus separating the HRV into low and high frequency (LF and HF) oscillations.^33^ The LF (0.04–0.15 Hz) HRV represents changes in heart rate attributed to baroreflex-mediated influences and reflect both sympathetic and parasympathetic activity.^34^ The HF HRV was set at an age-dependent threshold based on infant respiratory rates (0.4–1.5 Hz), to indicate changes in heart rate associated with respiratory activity and represent the fast-acting parasympathetic branch.^34,35^ The ratio between LF and HF HRV (LF/HF) indicates sympathovagal balance.^34^ HRV (LF, HF, and LF/HF) for each time window were calculated from the area under the power spectral density functions in each frequency band and represent the square of the amplitude of an oscillatory signal in the frequency range. The LF and HF are presented as absolute power, in ms^2^. The R–R interval was also used to calculate heart rate (HR, bpm) in the time windows. KMC was performed after feeds and no handling was performed during KMC.

The data was then manually viewed to remove any remaining artifacts. In addition, acute heart rate decelerations and transient bradycardic episodes frequently experienced by preterm infants markedly increase the HRV. Therefore, periods of acute bradycardia with HR falling to <80% of the baseline were identified and removed from the HRV and HR data.

### Statistical analyses

Data were checked for normality and statistically analysed using GraphPad Prism (version 10.1.2; GraphPad Software, CA, United States). Clinical characteristics were compared between the FGR and AGA groups with an unpaired Student *t* test, or the Fisher’s Exact test for proportions. The unpaired Student *t* test, or the Mann–Whitney test for nonparametric data was used to compare HR, SpO_2_, HRV (LF, HF and LF/HF) and total duration of acute bradycardia between FGR and AGA infants at baseline and during KMC respectively. HR, SpO_2_, HRV parameters and total duration of acute bradycardia were compared between the baseline (pre-KMC period) and during KMC using paired Student *t* test or the Wilcoxon test for non-parametric data. Comparison of HR and HRV parameters between the baseline and KMC were also performed for infants with baseline HRV above and below the median in each group respectively, using Student *t* test or the Wilcoxon test for non-parametric data. The Fisher’s Exact test was used to compare proportion of infants with an increase in HRV parameter during KMC that is more than 2 standard deviations (SD) of the individual baseline. Clinical characteristics of infants are presented as mean ± SD. HRV parameters, HR, duration of bradycardic episodes and SpO_2_ are presented as mean with 95% confidence interval.

## Results

Twenty-two preterm FGR infants and twenty AGA were studied on or after postnatal day 8. All but one preterm FGR infants were delivered via emergency caesarean section due to abnormal cardiotocography, and/or worsening foetal multi-vessel integrated Doppler measurements, and/or severe maternal pre-eclampsia. The remaining preterm FGR infant received no antenatal care due to a concealed pregnancy, and the FGR was diagnosed postnatally based on weight.

Clinical characteristics of the cohort are summarised in Table 1. By design, birth weight and weight at study, along with z-scores, were significantly lower in the FGR group compared to AGA counterparts (*P* < 0.05 for all). There are no significant differences between the FGR and AGA groups in gestational age at birth, sex, postmenstrual or postnatal age at time of study and respiratory support during study.

**Table 1:** Clinical characteristics of the infant population.

	FGR	AGA
Number of infants, *n*	22	20
GA at birth, *weeks*	30.0 ± 2.0	29.1 ± 1.9
Sex, *male/female*	9/13	8/12
Birthweight, *grams*	877 ± 292***	1263 ± 329
Birthweight, *z-score*	−1.68 ± 0.40***	0.14 ± 0.84
APGAR *score*	8 ± 2	8 ± 1
Age at Study		
‐ Postnatal age, *days*	15.4 ± 6.9	15.9 ± 6.5
‐ Postmenstrual age, *weeks*	32.0 ± 1.4	31.2 ± 1.5
Weight at study, *grams*	1007 ± 271***	1368 ± 294
Weight at study, *z-score*	−1.98 ± 0.461***	−0.64 ± 0.65
Respiratory support during study, *n*		
‐ None	9	11
‐ High Flow	0	1
‐ Continuous positive airway pressure	13	8

Values are in mean ± standard deviation unless otherwise stated.

*FGR* foetal growth restriction, *AGA* appropriate for gestational age, *GA* gestational age.

***Indicates statistically significant difference between FGR and AGA infants (*p* < 0.001).

Table 2 provides the heart rate, HRV parameters, and the total duration of bradycardic episodes before and during KMC. HR was lower during KMC compared to baseline for the entire group (*P* < 0.05). There was no statistically significant difference in all other parameters during the KMC compared to the baselines for the entire group, FGR or AGA groups. No difference was found between FGR and AGA infants in all parameters at baseline or during KMC.

**Table 2:** Cardiorespiratory parameters and power of HRV at baseline and during KMC.

		ALL (*N* = 42)	FGR (*N* = 22)	AGA (*N* = 20)
Heart rate, *bpm*	Baseline	162.8 (159.2, 166.4)	161.1 (155.9, 166.4)	164.6 (159.3, 169.9)
	KMC	160.0 (156.6, 163.4)*	157.0 (151.6, 162.3)	163.3 (159.2, 167.4)
Total duration of bradycardic episodes, *s*	Baseline	19.1 (3.7, 34.4)	11.1 (−6.4, 28.5)	27.9 (0.6, 55.1)
	KMC	24.72 (13.2, 36.2)	14.1 (2.5, 25.7)	36.4 (16.0, 56.7)
SpO_**2**_, %	Baseline	97.05 (96.38, 97.72)	96.79 (95.74, 97.84)	97.33 (96.45, 98.22)
	KMC	96.81 (96.13, 97.49)	96.43 (95.34, 97.52)	97.23 (96.4, 98.06)
LF	Baseline	30.1 (21.6, 38.7)	26.9 (15.5, 38.4)	33.6 (19.9, 47.4)
	KMC	29.4 (21.0, 37.7)	26.9 (16.5, 37.4)	32.0 (17.7, 46.4)
HF	Baseline	9.4 (6.3, 12.6)	7.9 (5.0, 10.8)	11.1 (5.0, 17.3)
	KMC	8.4 (5.7, 11.0)	7.6 (4.1, 11.2)	9.2 (4.9, 13.5)
LF/HF	Baseline	3.7 (2.9, 4.6)	3.9 (2.5, 5.4)	3.5 (2.6, 4.4)
	KMC	4.0 (3.2, 4.8)	4.1 (2.7, 5.4)	3.9 (2.9, 4.8)

All values are expressed as mean (lower 95% CI, upper 95% CI).

*CI* confidence interval, *HRV* heart rate variability, *KMC* kangaroo mother care, *LF* Low Frequency HRV, *HF* High Frequency HRV, *LF/HF* LF to HF ratio.

*Indicates statistically significant difference between baseline & KMC (p < 0.05).

**Fig. 1:**
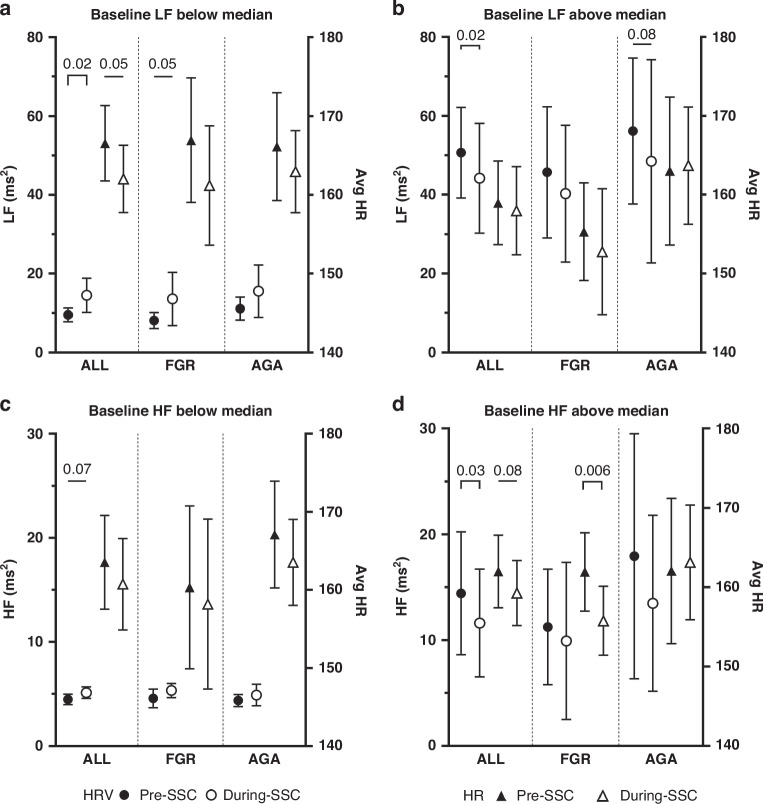
Effect of kangaroo mother care (KMC) on heart rate (HR) and heart rate variability (HRV) spectral indices. HR and HRV in the low-frequency (LF) (**A**, **B**) and high-frequency (HF) (**C**, **D**) ranges, according to the infants’ baseline HRV and size at birth, are shown. The HR and HRV before KMC are represented by the closed triangles and circles; the HR and HRV during KMC by the open triangles and circles. Data are represented as mean ±95% confidence interval.

Infants were divided into those with baseline HRV above and below the median in the entire group, FGR and AGA group. Figure 1 illustrates the effect of KMC on HRV, according to the baseline HRV. For the entire group, infants with low baseline LF below the median had increased HRV during KMC (*p* = 0.02, Fig. 1a) with a reduced HR (*p* = 0.05). A similar trend was noted in infants with low baseline HF below the median, though the increase in HF during KMC did not reach statistical significance (*p* = 0.07, Fig. 1c). In contrast, infants with high baseline HRV above the median had decreased HRV during KMC, both at LF and HF ranges (*p* = 0.02 and *p* = 0.03 respectively, Fig. 1b, d).

Analyses was then performed for the FGR and AGA infants respectively. FGR infants with low baseline LF below the median had increased HRV during KMC (*p* = 0.05, Fig. 1a). In the AGA group, no difference in HRV parameters was found during KMC regardless of the baseline HRV. LF/HF ratio showed no significant changes during KMC across study groups.

HR overall decreased during KMC regardless of baseline HRV values and reached significance in all infants with low baseline LF (Fig. 1a) and FGR infants with high baseline HF (Fig. 1d).

Table 3 summarises the proportion of infants with an increase in HRV during KMC that is more than 2 standard deviations (SD) of the individual baseline data. For the entire group, a higher proportion of infants with low baseline LF showed increase in HRV of >2 SD (6/21), compared to infants with high baseline LF (0/21, *p* = 0.02). No significant differences were identified in either FGR or AGA groups, or in the HF and LF/HF analyses.

**Table 3:** Proportion of infants with an increase in HRV during KMC that is >2 standard deviations (SD) of the individual baseline.

		Infants with baseline HRV below median	Infants with baseline HRV above median
LF	ALL	6/21*	0/21
	FGR	4/11	0/11
	AGA	2/10	0/10
HF	ALL	4/21	1/21
	FGR	3/11	1/11
	AGA	1/10	0/10
LF/HF	ALL	0/21	1/21
	FGR	0/11	1/11
	AGA	0/10	0/10

All values are number of infants.

*HRV* heart rate variability, *LF* Low Frequency, *HF* High Frequency, *LF/HF* LF to HF ratio ALL, all infants, *FGR* foetal growth restriction, *AGA* appropriate for gestational age.

*Indicates statistically significant difference between the infants with baseline HRV below median vs above median (*p* < 0.05).

## Discussion

To our knowledge, this is the first study to investigate the impact of KMC on autonomic cardiovascular control in preterm FGR infants. We have shown that preterm infants with low baseline HRV are more likely to increase their HRV during KMC, an effect that appears stronger in FGR infants compared to their AGA counterparts.

The interpretation of changes to HRV in adults has been well studied. Decreased HRV (either LF or HF) is associated with greater cardiovascular mortality and morbidity in adults, such as hypertension and diabetes.^36–40^ Further, pain and anxiety reduce HF, indicating decreased parasympathetic activity due to unpleasant stimuli.^41,42^ Interpretation of HRV in preterm neonates involve further considerations compared to adults. The sympathetic branch develops most rapidly in the first trimester, whereas vagal parasympathetic control matures later in foetal development, thus preterm birth predominantly affects parasympathetic maturation.^43^ Accordingly, preterm birth is associated with reduced HRV, with a marked reduction in HF in those born at the earliest gestational ages.^44^ In addition, preterm infants have lower HRV compared to term infants at term-equivalent age, suggestive of delayed maturation of the autonomic nervous system.^45,46^ Notably, preterm infants display a larger variation in HR and respiratory rate; and are prone to acute tachycardia and bradycardia, for example, apnoea of prematurity causes acute bradycardia and oxygen desaturations.^24^ These rapid HR changes transiently increased HRV and may reflect clinical instability due to immature cardiorespiratory control or autonomic disturbances due to external stimuli.^24,47^ We therefore excluded transient periods of bradycardias to avoid the potential confounding effect on HRV.

With increasing postmenstrual age, HRV increases gradually, consistent with autonomic maturation, improved ‘fight or flight’ response to external stimuli and ability to maintain homoeostasis.^45,48^ As KMC provides physiological stability and therefore would be expected to promote autonomic control, we hypothesised that KMC would lead to increased HRV. Nonetheless, existing literature regarding the effect of KMC on HRV yields mixed results. A crossover study on 14 preterm infants showed no significant difference in either LF or HF during KMC compared to an incubator environment, although gestationally older infants displayed higher HRV as expected.^48^ In a single-patient case study on a preterm infant, both LF and HF decreased as the infant’s activity progressed from being fussy at baseline to a period of sleep during KMC.^47^ In contrast, a study on 14 preterm infants demonstrated increased LF and HF during KMC compared to the incubator, which were attributed to physiological impact of maternal presence and touch.^49^ Another study also described an increased LF, however with decreased HF during KMC compared to baseline in 16 preterm infants.^50^ Interestingly, preterm infants showed predominantly increased LF compared to HF when they were placed in a 30° head-up tilt position from being supine, similar to that during KMC.^51^

### LF HRV

We observed that infants with low LF at baseline showed increased HRV and decreased HR during KMC, suggesting positive effects from KMC with improved autonomic control; and this impact was stronger in the FGR infants. Previous work by others and us show reduced HRV in FGR fetuses and preterm FGR infant on Day 1 of life compared with their AGA peers, consistent with compromised autonomic development due to the unfavourable intrauterine environment.^16–18,21^ The HRV difference between FGR and AGA infants was no longer detected at 1 month postnatal age,^21^ consistent with our current results in preterm infants at averaged two weeks of postnatal age. Several studies have demonstrated higher LF during KMC,^49,50^ and suggested this could be underpinned by increased sympathetic activities due to the infants’ head being in an up-tilting position during KMC with gravity causing pooling of blood, thereby activating baroreceptors.^51^ Infant sympathetic activity could also be increased due to maternal presence and touch as what occurs during mother-infant bed-sharing, and may be partly explained by thermal stimulation with the kangaroo mother contact.^52^ Notably, the reduced HR during KMC indicate that there were no excessive sympathetic activations. There was also no change in the LF/HF ratio, suggesting no predominance of sympathetic tone with the change to head-up position. Taken together, KMC leads to increased sympathetic regulation without causing sympathetic hyperactivity in infants with low baseline HRV, particularly in FGR infants.

### HF HRV

HF has been reported to decrease with pain, fear or anxiety in adults,^41^ and during surgery or noxious stimuli in infants and children,^53–55^ consistent with reduced parasympathetic activity following nociceptive stimuli. A few studies in preterm infants showed that HF indices increased during KMC, suggesting a positive impact of KMC with increased parasympathetic activities.^28,49^ In our study, infants with lower baseline HF showed a trend of higher HF during KMC, similar to a previous study which suggested that these infants with low baseline HF were less comfortable prior to KMC.^31^ The higher HF during KMC may be due to activation of pressure receptors that can increase parasympathetic activities, as HF and vagal tone in preterm infants were also increased by massage therapies^56,57^ and hand containment together with maternal voice.^58^

### Infants with high baseline HRV showed reduced HRV during KMC

On the other hand, infants with high baseline LF or HF had decreased HRV during KMC. Previous work showed that HRV parameters were reduced during KMC due to a decrease in transient HR decelerations.^24^ However, our analyses already excluded all periods of acute bradycardia. Our result is similar to a previous study that suggested the infants with high baseline HF were comfortable prior to KMC,^31^ and the transfer out of incubator could have disturbed the infant. However, our infants with high baseline HRV did not show increased HR during KMC to suggest reduced comfort.

We speculate the explanation may be related to the infants’ sleep state prior to and during the KMC. Preterm infants typically spend 80% of sleep time in active sleep at 29–30 weeks’ GA, which decreases to 55% by term-equivalent age.^59^ Both KMC and the prone position have been independently reported to increase the proportion of quiet sleep.^30,60^ HRV is lower during quiet sleep compared to active sleep.^61^ During KMC in the prone position, increase in quiet sleep may have decreased both the HRV and HR. However, this needs to be verified in future studies with sleep state assessment either by cotside behavioural scores and/or EEG.

Our study has several limitations. Our sample size was small, and the infants were stable clinically on non-invasive respiratory support with relatively few episodes of bradycardia and desaturations. This may explain why we found no significant impact of KMC on durations of acute bradycardia which were also not different between the FGR and AGA groups. The impact of KMC in the more unstable infants with cardiorespiratory dysfunction remains to be elucidated. In addition, measurements of blood pressure, temperature and cutaneous partial pressure of carbon dioxide, as well as neuromonitoring (EEG or near infra-red spectroscopy) would enhance the physiological insights of KMC. We did not score the infant sleep state, which could account for some of the changes in HRV. Future studies in infants with behavioural and sleep state scoring would be useful to further understand the impact of KMC on autonomic activities.

## Conclusion

We have shown that preterm infants with low baseline LF HRV are more likely to benefit from KMC and increase their HRV, suggesting improved autonomic control. This effect is stronger in infants born following FGR than their AGA counterparts.

## Data Availability

The datasets generated during and/or analysed during the current study are not publicly available due to human research ethics requirements but are available from the corresponding author on reasonable request.
